# Redo mitral valve replacement in an adult with severe pulmonary hypertension resulting from structural valve deterioration and left ventricular outflow tract obstruction and a history of atrioventricular septal defect repair: a case report

**DOI:** 10.1186/s13019-023-02371-6

**Published:** 2023-10-05

**Authors:** Kayo Sugiyama, Katsuhiko Matsuyama, Hitoshi Ogino

**Affiliations:** 1 Department of Cardiovascular Surgery, Tokyo Medical University Hospital, 6-7-1 Nishishinjuku, Shinjuku-ku, Tokyo 160-0023 Japan; 2 Department of Cardiac Surgery, Aichi Medical University Hospital, Nagakute, Japan

**Keywords:** Atrioventricular septal defect, Calcified bioprosthetic mitral valve, Pulmonary hypertension, Left ventricular outflow tract obstruction, Stent post protrusion

## Abstract

**Background:**

Pulmonary hypertension (PH)-associated with left heart disease (Nice PH classification group II) improves when the latter is treated; however, the treatment of PH concomitant with group I PH due to congenital heart disease is difficult, and the optimal pharmacotherapy is controversial. Intervention strategies for the left-sided atrioventricular valve in partial atrioventricular septal defect (AVSD) are problematic.

**Case presentation:**

A 37-year-old woman who had undergone patch closure for a partial AVSD and mitral valve replacement with a rather large bioprosthesis at the juxta-annular position for mitral regurgitation 12 years earlier was referred to our institute because of severe PH. Echocardiography revealed calcification resulting in severe stenosis of the bioprosthesis and protrusion of its stent post into the left ventricular outflow tract; therefore, redo mitral valve replacement at the supra-annular position was performed using a mechanical valve. Combined group I and II PH gradually improved with meticulous postoperative medical management.

**Conclusions:**

Severe PH due to stent post protrusion and structural valve deterioration in AVSD was successfully treated with redo mitral valve replacement. The present case was complicated with group I and II PH, for which medical therapy in conjunction with surgical treatment yielded an optimal therapeutic effect.

## Background

Structural valve deterioration due to severe calcification of the mitral valve bioprosthesis can cause severe pulmonary hypertension (PH). Surgery is more hazardous in these patients compared to those with a lower pulmonary arterial pressure (PAP) [1–3], while definitive surgery is the only solution, leading to a therapeutic dilemma. Although elongation and narrowing of the left ventricular outflow tract (LVOT) in patients with atrioventricular septal defect (AVSD) is a well-known phenomenon [4, 5], appropriate therapeutic strategies for coexisting left-sided atrioventricular valve disease in adults with AVSD have not been sufficiently discussed. To prevent left ventricular outflow tract obstruction (LVOTO) in cases of mitral valve replacement, appropriate size and orientation of the prosthesis are vital [6, 7] and in the current era, preservation of more left-sided atrioventricular valves can be ensured, with excellent early and long-term survival and acceptable reoperation rates [8].

## Case presentation

This study complied with the principles of the Declaration of Helsinki. The patient provided written informed consent for the use of her clinical data for scientific presentations or publications.

A 37-year-old woman with severe PH was referred to our institute for redo mitral valve replacement. Although she was diagnosed with partial AVSD at the age of 2 years, she grew up without critical symptoms [New York Heart Association (NYHA) Class II]. At the age of 25 years, she underwent the following surgical treatment at another hospital: patch closure for the partial AVSD, mitral valve replacement using a 29-mm Carpentier-Edwards Perimount bioprosthesis (Edwards Lifesciences, Irvine, CA, USA) in the juxta-annular position for moderate-to-severe mitral valve regurgitation, and tricuspid suture annuloplasty using Kay’s technique and implantation of epicardial pacing leads for moderate-to-severe tricuspid valve regurgitation. Subsequently, a pacemaker generator was implanted in the subcutaneous space of the upper abdomen for the complete atrioventricular block. Since her body surface area was 1.33 m/s^2^, the mitral valve prosthesis was large. Three years prior to admission, she developed dyspnea on exertion (NYHA Class III) and was referred to our institute for severe PH at the age of 37 years, 12 years after the previous surgery.

**Fig. 1:**
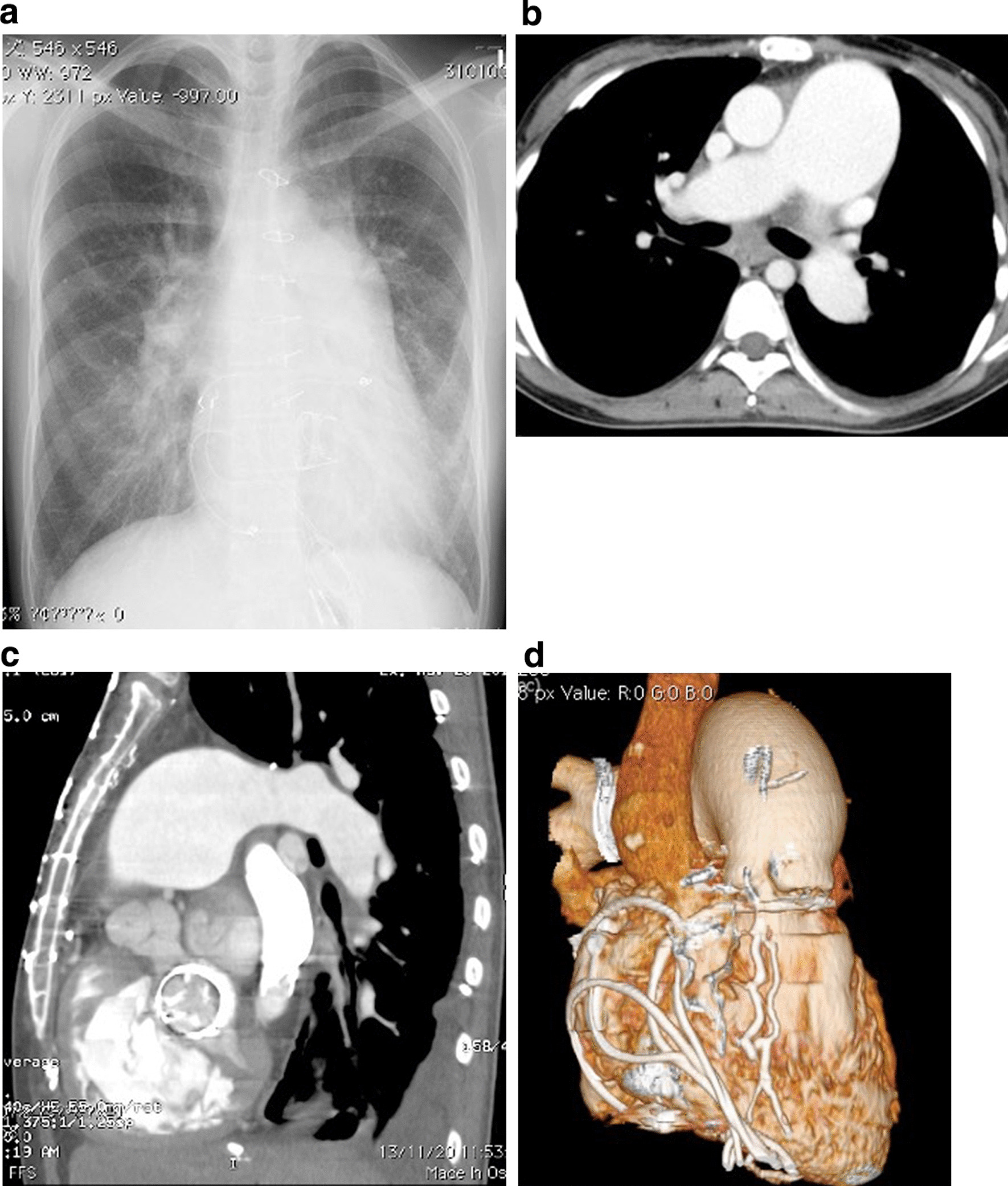
**a**. Preoperative chest radiograph shows cardiomegaly involving the right cavities and notable enlargement of the pulmonary artery. **b** Preoperative computed tomography of the lung shows notable enlargement of the pulmonary artery. **c** Preoperative computed tomography of the lung shows notable enlargement of the pulmonary artery and severe calcification of the bioprosthesis. **d** Preoperative computed tomography of the lung shows notable enlargement of the pulmonary artery and severe adhesion of the previous pacing wires in the right atrium

**Fig. 2:**
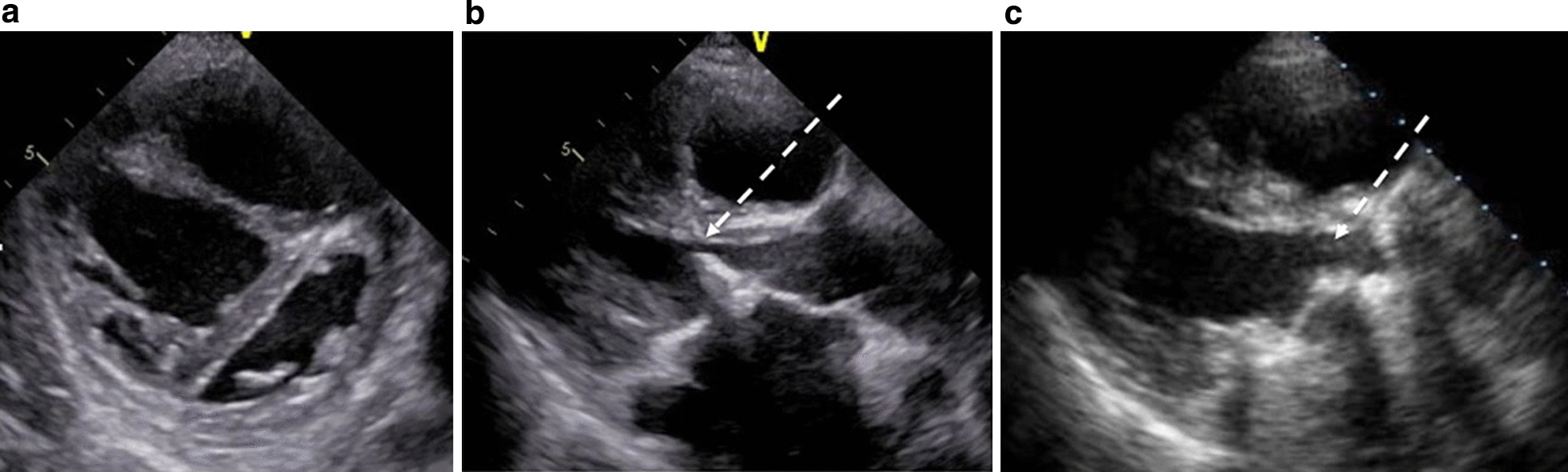
**a** Preoperative echocardiography shows a small left ventricle being compressed by a dilated right ventricle. **b** Preoperative echocardiography shows left ventricular outflow tract obstruction due to stent-post protrusion of the bioprosthetic mitral valve (dotted arrow). **c** Postoperative echocardiography shows improvement of left ventricular outflow tract obstruction (dotted arrow)

The preoperative laboratory data indicated heart failure, with N-terminal pro-brain natriuretic peptide levels of 993 pg/mL and percutaneous oxygen saturation of 94% at room air. Chest radiography revealed cardiomegaly involving the right cavities and prominent enlargement of the pulmonary artery (Fig. 1a). Computed tomography also showed notable enlargement of the pulmonary artery (Fig. 1b), accompanied by severe calcification of the bioprosthesis (Fig. 1c) and severe adhesion of the previously placed pacing wires in the right atrium (Fig. 1d). Transthoracic echocardiography revealed a small left ventricle compressed by a dilated right ventricle (Fig. 2a) and severe PH, with an estimated right ventricular pressure of 112 mmHg. Echocardiography also revealed LVOTO accompanied by severe calcification due to protrusion of the stent post of the mitral bioprosthesis (Fig. 2b). The maximum velocity and pressure gradient through the LVOT were 2.9 m/s and 26 mmHg, respectively. The maximum velocity and pressure gradient through the prosthetic mitral valve were 2.7 m/s and 20 mmHg, respectively, and the area of the prosthetic mitral valve’s orifice was 0.68 cm^2^.

Right heart catheterization revealed features indicative of severe PH with the following parametric values: PAP, 104/43 mmHg (mean, 64 mmHg), which was equal to the systemic arterial blood pressure; calculated total pulmonary resistance (TPR), 1462 dyne/s/cm^5^, and pulmonary vascular resistance (PVR), 891 dyne/s/cm^5^. Initially, pulmonary arterial hypertension (group I PH) was considered due to the patient's long-term history of congenital heart disease; however, coexisting group II PH was also considered. Therefore, low-dose prostaglandin I_2_ was administered preoperatively for the treatment of PH, owing to concerns of pulmonary congestion. Since PH exceeding 100 mmHg was considered to be caused by LVOTO due to stent-post protrusion and severe calcification of the mitral bioprosthesis, surgical repair of the redo mitral valve replacement was planned.

**Fig. 3:**
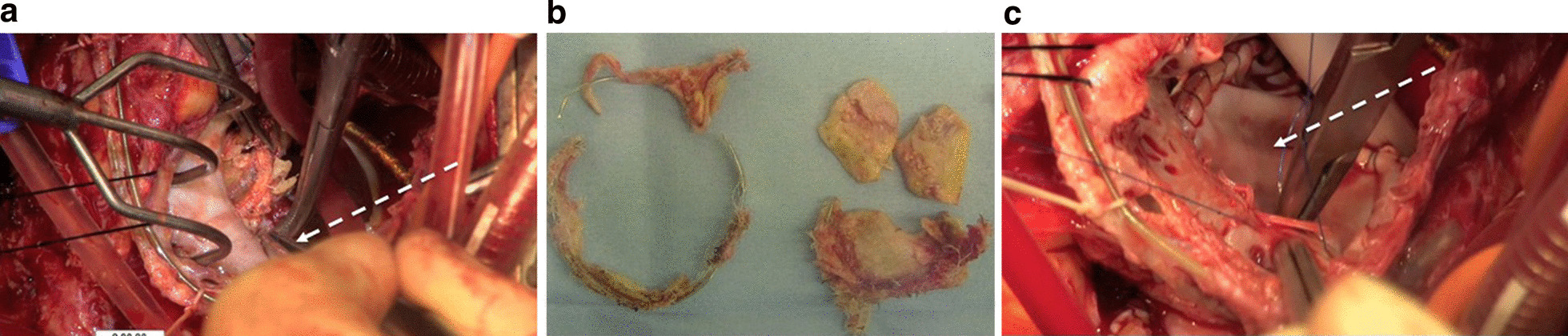
**a** Intraoperative view shows left ventricular outflow tract obstruction due to stent post protrusion of the bioprosthetic mitral valve; the tip of the forceps slightly passes through the left ventricular outflow tract (white dotted arrow). **b** Intraoperative view shows thickened leaflet indicating structural valve deterioration of the bioprosthesis; left: ring of the prosthetic valve, right: thickened and calcified valve leaflet of the prosthetic valve. **c** Intraoperative view shows reconstruction of the mitral annulus with bovine pericardium; reinforced bioprosthetic valve and closure of the atrial septal defect with a bovine pericardial patch (white dotted arrow)

Redo mitral valve replacement was performed through a median re-sternotomy. Marked dilatation of the pulmonary artery and severe adhesion of the previous pacing wires to the right atrium were observed. After careful dissection of the adhesions, cardiopulmonary bypass was instituted via bicaval drainage and ascending aortic cannulation. As retrograde cardioplegia was impossible owing to the patency of the left superior vena cava, cardiac arrest was achieved using antegrade cardioplegia, and the mitral valve prosthesis was exposed through the left atrium. Eventually, the patent left superior vena cava was found to be connected to the left atrium; therefore, a drainage cannula was placed in the coronary sinus. There were concerns about a small amount of residual shunt blood flow; however, given the high degree of adhesion, we did not reconstruct the abnormal connection between the left superior vena cava and left atrium. Severe stenosis in the LVOT due to stent-post protrusion (Fig. 3a) and severe calcification of the mitral bioprosthesis (Fig. 3b) were observed. Since the LVOT was narrowed and the native aortic valve was close to the juxtapositioned mitral bioprosthesis in a heart with AVSD, removal of the previous prosthetic mitral valve was quite difficult. The protruding strut was highly adherent to the LVOT, and accidental injury to the noncoronary aortic cusp was identified through antegrade cardioplegia. Redo mitral valve replacement in the supra-annular position was performed using a 25-mm SJM Regent mechanical valve (St. Jude Medical, St. Paul, MN, USA), along with annular plication using a bovine pericardium collar (Fig. 3c). Aortic valve repair seemed difficult because the cusp was extremely vulnerable and fragile, and was eventually replaced. Our first choice, a 17-mm SJM Regent mechanical valve, was impossible to seat in the small annulus; thus, we used a 16-mm ATS-AP mechanical valve (Medtronic, Minneapolis, MN, USA), which was placed in a tilted position. Since the ascending aortic wall was also vulnerable, closure was achieved with two-layered mattress sutures with 5–0 prolene. Subsequently, we performed tricuspid valve annuloplasty for severe tricuspid regurgitation using a 27-mm SJM Tailor band (St. Jude Medical) and closure of the atrial septal defect with a bovine pericardial patch after removal of the old calcified patch. Weaning from cardiopulmonary bypass was achieved with the administration of inotropics and low-dose prostaglandin I_2_; however, the PAP remained high at 60% of systemic pressure. Extubation was achieved on the second day of surgery with high-dose inotropics and oxygen, followed by the administration of oral bosentan hydrate, in addition to anticoagulant therapy. Postoperative echocardiography showed improvement in the LVOTO (Fig. 2c) and PH with an estimated right ventricular pressure of 48 mmHg. The maximum velocity and pressure gradient between aortic valve and left ventricle were 1.9 m/s and 5 mmHg, respectively. The maximum velocity and pressure gradient through the prosthetic mitral valve were 1.8 m/s and 4 mmHg, respectively. Since the mechanical valve in the aortic valve position was small for the patient’s body size, the maximum velocity through the aortic mechanical valve was 3.1 m/s, the peak and mean pressure gradient were 38 and 20 mmHg, respectively, even without obvious symptoms of heart failure.

Postoperative right heart catheterization revealed a mean PAP of 43 mmHg, calculated TPR of 937 dyne/s/cm^5^, and PVR of 643 dyne/s/cm^5^. Subsequently, the patient was discharged on postoperative day 43 after sufficient rehabilitation and meticulous pharmacotherapeutic administration, but the requirement of home oxygen therapy for hypoxia was maintained; percutaneous oxygen saturation was 92% under an oxygen supply of 2 L/min. Bosentan hydrate was administered for 2 months postoperatively. Moreover, digoxin was administered postoperatively, which was discontinued 4 years postoperatively and replaced with bisoprolol fumarate. The dose of the diuretic, i.e., furosemide 40 mg was tapered to 10 mg. Two years postoperatively, her oxygen level improved to almost within the normal range, and home oxygen therapy was discontinued; percutaneous oxygen saturation gradually improved to 96% at room air, and her PAP decreased to almost within the normal range, with a systolic PAP of 26 mmHg. The patient continues to do well (NYHA class II), without any major adverse cardiovascular events after 6 years of follow-up.

## Discussion

After tissue valve replacement, calcification of bioprostheses fabricated from glutaraldehyde-cross-linked porcine aortic valves or bovine pericardium frequently is responsible for clinical failure of these devices [9]. Severe calcification of the mitral valve bioprosthesis can lead to severe PH, which can become clinically problematic. Although mitral valve repair yields a poor prognosis in such cases [1–3], medical therapy is ineffective, while corrective surgery offers definitive treatment. Since it was initially unclear whether the present patient had group I or group II PH, and pulmonary congestion was a concern, low-dose prostaglandin I_2_ was administered preoperatively. After repair of left-sided heart disease, meticulous treatment with bosentan hydrate and home oxygen therapy was continued until PH and percutaneous oxygen saturation improved. Thus, a multidisciplinary approach is needed for the treatment of patients with groups I and II PH.

Bioprostheses implanted in the mitral position can reportedly cause LVOTO [6, 7, 10, 11], especially in patients with a narrowed mitral-aortic angle, thickened interventricular septum, systolic anterior motion, small LV cavity, and atrial fibrillation [6, 10, 12]. The stent post of the bioprosthesis tends to protrude higher than the mechanical valve [7, 10, 11]; however, PH due to stent-post protrusion of the bioprosthetic mitral valve into the LVOT is rare [11, 13, 14]. In the present case, juxta-annular orientation of the 29-mm Carpentier-Edwards Perimount bioprosthesis, which seemed to be too large for the patient’s body size, and AVSD might have caused worsening of the coexisting LVOTO. Low-profile prostheses or mechanical valves in the supra-annular position are recommended to prevent LVOTO in patients with isolated mitral disease and a small LV cavity [7].

AVSD is characterized by defects in the AV septum and adjacent atrial and ventricular septa, prolongation and narrowing of the LVOT, and malformation of the AV valves with a common AV junction [4]. Iatrogenic subaortic narrowing occurs when surgical correction of the left AV valve is performed without sufficient correction of the “scooped-out” septum caused by ventricular septal extension [5]. Moreover, modified single-patch AVSD repair is associated with a higher frequency of reoperation for LVOTO [15]. Thus, the present patient presented with strong risk factors for LVOTO. Surgical techniques for left-sided atrioventricular valve repair in hearts with AVSD have been improved and standardized, building on experience from mitral valve repair techniques [8]. In the present case, repair for mitral valve regurgitation should have been considered initially. The velocity through the small mechanical aortic valve should also be considered in the future.

## Conclusions

The occurrence of LVOTO should be considered in adult patients with AVSD, which may be prevented by mitral valve repair. Pulmonary hypertension complicated by left-sided heart disease in an adult with congenital heart disease should be carefully managed with optimal medical therapy in addition to surgical correction.

## Data Availability

All data generated or analyzed during this study are included in this article.

## Abbreviations

AVSD: Atrioventricular septal defect
LVOTO: Left ventricular outflow tract obstruction
MVR: Mitral valve replacement
PAP: Pulmonary arterial pressure
PH: Pulmonary hypertension
PVR: Pulmonary vascular resistance
TPR: Total pulmonary resistance
